# Editorial: Polymicrobial Biofilms in Chronic Infectious Disease

**DOI:** 10.3389/fcimb.2020.628584

**Published:** 2020-12-21

**Authors:** Freya Harrison, Raymond N. Allan, Sarah E. Maddocks

**Affiliations:** ^1^School of Life Sciences, The University of Warwick, Coventry, United Kingdom; ^2^Health and Life Sciences, Leicester School of Pharmacy, De Montfort University, The Gateway, Leicester, United Kingdom; ^3^School of Sport and Health Sciences, Cardiff Metropolitan University, Cardiff, United Kingdom

**Keywords:** biofilm, microbial community, ecosystem, virulence, microbial diversity, infection model, biofilm disruption

Polymicrobial biofilms are associated with chronic infections of various sites in the body. Interactions among the host microbiota and colonizing pathogens are thought to be important for the establishment of a persistent, chronic infection community and the resilience of this community in the face of host immune defences and medical intervention. The view of polymicrobial infection biofilms as dynamic, evolving ecosystems has become dominant among microbiologists, especially with regard to particularly well-studied infection contexts such as periodontitis, cystic fibrosis lung infections and non-healing wounds. This has led to suggestions that improved treatments for polymicrobial biofilm infections might rely on targeting ecological interactions between bacterial species or strains which are fundamental to the ecosystem functioning of the biofilm, or to the expression of highly virulent or persistent phenotypes by its members.

The reviews by Kvich et al. and Welp and Bomberger frame our current understanding of microbial diversity in a range of chronic infection contexts and highlight the need for a better understanding of polymicrobial community and interactions. Welp and Bomberger provide a detailed dissection of the various interactions which have been proposed or reported to occur between microbes in chronic respiratory disease, and the potential for these to affect patient outcomes. Kvich et al. broaden the focus to discuss how clinicians and researchers seek evidence for mixed-species biofilm at infection sites, and provide an important cautionary note that clear evidence for the microgeography of biofilms from chronic infection sites is a crucial foundation to identifying those inter-bacterial interactions which are truly relevant *in vivo*: potential synergistic or antagonistic interactions between co-isolated microbes may not occur *in vivo* if the microbes in question form spatially-segregated communities within the biofilm.

The need to understand “who is where?” in biofilms from polymicrobial infections goes hand in hand with a need to identify which bacterial interaction phenotypes are expressed in specific chronic infection contexts. Three of the primary research papers in this collection ask mechanistic questions about inter-bacterial and inter-kingdom interactions in biofilm infection. Collectively, these studies describe the mechanisms underpinning new and known interspecies interactions that are fundamental to co-infection scenarios in chronic lung, wound, and periodontal disease. While focus is predominantly placed on understanding the role of key virulence factors, we also should not ignore other cellular features or pathways that may be important in polymicrobial biofilm development and survival. This is exemplified by the research of Ng et al. showing that, in addition to mobility-related pore formation within the biofilm architecture, the periplasmic flagella of *T. denticola* have a wider-reaching synergistic influence on biofilms formed in conjunction with *P. gingivalis*.

An important approach that would better facilitate our understanding of these complex interactions, and expedite the development of tailored therapeutics for polymicrobial infections, is through the utilization of databases similar to that developed by Grainha et al. The “Inter-species CrossTalk Database” is a valuable resource containing curated experimental data that allows researchers to interrogate the interactions between *P. aeruginosa* and *C. albicans*, two frequently co-isolated species in polymicrobial infections. This research also serves to highlight the importance experimental conditions play in influencing the dynamic interplay between different members of the microbial community.

Therefore, in order to translate this knowledge into effective management strategies for the treatment of biofilm infections, realistic infection modeling is key. Phan et al. present a novel chronic wound model that combined with analysis of volatile organic compounds reveals that environmental stresses in this environment favor the abundant growth of *P. aeruginosa*. In addition to revealing much about microbial community interactions, such models provide a robust means for developing effective strategies to disrupt biofilm growth. This is also typified by the use artificial sputum medium to better simulate the physiochemical conditions of the CF lung. This model was used by Silva et al. to demonstrate that treatment of polymicrobial biofilms (*P. aeruginosa*, *S. aureus*, and *B. cenocepacia*) using aspartic acid or succinic acid as adjuvants improves the efficacy of ciprofloxacin in removing *P. aeruginosa*. The impact of environment on the effectiveness of biofilm dispersants is also demonstrated by Redman et al. where the capacity of two glycoside hydrolases against *P*. aeruginosa and *S. aureus* in murine and *in vitro* models was examined. Their results show that the enzymes perform differently in different models, and in mono vs. dual-species biofilms. Significantly, the environment afforded by each type of model appeared to impact the constituents of the EPS. This serves to reiterate the importance of understanding how best to target microbial communities in different infection contexts.

This themed collection of original review and research articles provides an overview of the importance of polymicrobial biofilms in chronic infections. In particular, they highlight the need for a multi-faceted approach that incorporates both the use of online databases for polymicrobial interactions, advanced imaging approaches, and the development of more biologically-relevant models to better understand the interplay between species. The ultimate aim of these models being to inform the development of much-needed novel therapeutics for these chronic infections.

## Author Contributions

All authors contributed to the article and approved the submitted version.

## Conflict of Interest

The authors declare that the research was conducted in the absence of any commercial or financial relationships that could be construed as a potential conflict of interest.

